# Evidences for a shared history for spectacled salamanders, haplotypes and climate

**DOI:** 10.1038/s41598-018-34854-1

**Published:** 2018-11-07

**Authors:** Mattia Iannella, Paola D’Alessandro, Maurizio Biondi

**Affiliations:** University of L’Aquila, Department of Health, Life, and Environmental Sciences, L’Aquila, 67100 Italy

## Abstract

The so-called glacial refugia, formed during the Pleistocene climatic oscillations, played a major role in shaping the distribution of European species, triggering migrations or isolating populations. Many of these events were recently investigated by genetic data, mainly for the European Last Glacial stage, in the Iberic, Italian and Greek-Balkan peninsulas. The amphibian genus *Salamandrina*, the most ancient living salamandrid lineage, was widespread in Europe until the climatic oscillations of Miocene probably forced it to shelter in the only suitable territory at that time, the Apennines. Nowadays this genus is endemic of peninsular Italy with two parapatric species, *S*. *perspicillata* and *S*. *terdigitata*, sharing an area of secondary contact formed after the Last Glacial Maximum. Climate is generally identified as the key factor for the interpretation of genetic data. In this research, we directly measure climate influences on the two *Salamandrina* known species through Ensemble Modelling techniques and post-modelling GIS analyses, integrating updated genetic data in this process. Our results confirm the hypotheses of southwards (and subsequent northwards) shifts, identify glacial refugia and corridors used for the post-glacial re-colonization. Finally, we map a contact zone deserving more sampling effort to disentangle the introgression and hybridization observed.

## Introduction

The distribution of a species is the result of different environmental factors, usually classified in biotic interactions, abiotic variables and dispersal capability/geographic accessibility, acting together and defining a certain area actually inhabited by the species^1^. Climate is one of the main abiotic variables which exerts a heavy influence over the environmental requirements of species, shaping their distribution in space and time^2–4^. The recent history of species living in temperate zones, for instance, was deeply conditioned by Pleistocene glaciations, which caused shifts in species’ ranges^5–7^. As a consequence, the so-called ‘refugia-within-refugia’ (*sensu* Gómez and Lunt)^8^ were formed in Europe during the last glacial stage^9–11^, especially in the Iberian, Italian and Greek peninsulas^8,10,12–14^. To better understand and describe the biogeographical phenomenon during Pleistocene cycles of glaciations, many researches focused on the amphibians, because of their sensibility to climatic variations, strict habitat requirements and relatively low vagility^15,16^. In the Italian peninsula, some studies were carried out over amphibians, in terms of genetic analyses, to mainly address the phylogenetic network and evolutionary history and recent demographic trends^17–19^. The two species belonging to the Italian endemic genus *Salamandrina* Fitzinger, 1826 (Amphibia: Caudata: Salamandridae)^20^, namely the Northern spectacled salamander *S*. *perspicillata*^21^ and the Southern spectacled salamander *S*. *terdigitata*^22^, were also studied in recent years^15,23–25^. They were identified as two distinct species through nuclear and mitochondrial data^26–28^, considering the lack of morphological distinctive traits, and that morphometry itself may fail to exactly discriminate the two species^29^. Based on genetic data, two glacial refugia, located in southern Latium (central Italy) for *S*. *perspicillata* and in Calabria for *S*. *terdigitata*, were found^15^. These hypotheses date back and are attributed to the climatic conditions of Last Glacial Maximum and the subsequent de-glaciation period, in which the two *Salamandrina* species expanded in the Italian peninsula, considering the more suitable climatic conditions^15^. Notwithstanding the interesting results obtained from these analyses about the heavy influence of climate over the two species, there are no researches which directly measure and infer past and current potential distributions based on climate itself, which is the aim of the present contribution. In fact, considering the current distribution of the two *Salamandrina* species, together with climatic data, we built Species Distribution Models (SDMs) and performed post-modelling cartographic analyses in GIS environment, to infer areas potentially suitable for the two *Salamandrina* species in past climatic conditions. We therefore propose a combined approach considering the information by the haplotype network, as proposed by Mattoccia, *et al*.^15^, and the predicted changes for the past habitat suitable areas in the distribution of *S*. *perspicillata* and *S*. *terdigitata*, integrating SDMs and genetic information into a single global model.

## Results

### Model performance and environmental suitability

**Figure 1:**
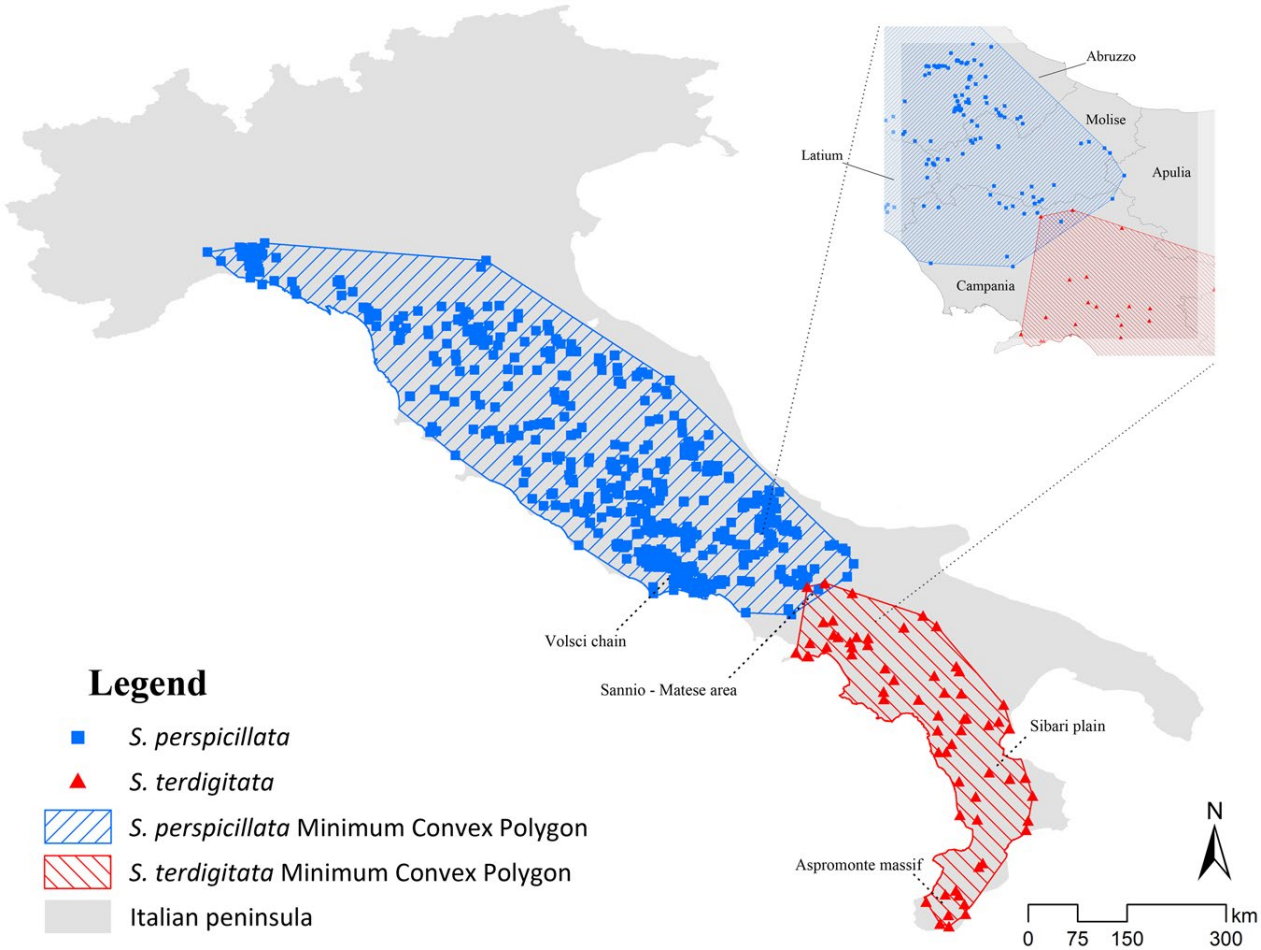
Study area and distribution of the two target species. The localities of *Salamandrina perspicillata* (blue squares) and *S*. *terdigitata* (red triangles) are mapped over the study area (grey). The Minimum Convex Polygons calculated over these two datasets are also reported, with the species’ respective colors.

For *Salamandrina perspicillata*, the thinning process resulted in 298 records out of the 779, with Moran’s I = −0.010 (expected value = −0.005), z-score = −0.223 and p = 0.823, while no thinned records (54 localities) were found for *S*. *terdigitata*, with Moran’s I = −0.150 (expected value = −0.022), z-score = −1.398 and p = 0.162, thus obtaining no spatial autocorrelation for both the analyzed target species’ datasets. The Minimum Convex Polygons built on the target species’ datasets cover the whole Apennine chain and show an overlap in the Sannio-Matese area, on the border between Campania and Molise regions (Fig. 1).

Multicollinearity among environmental predictors was avoided by discarding seven variables, on the basis of the correlation matrix reported in Supplementary Table S1 (variables highlighted in yellow). The models were then calibrated with the following twelve variables: BIO1 (annual mean temperature), BIO2 (mean diurnal range), BIO3 (isothermality), BIO6 (minimum temperature of coldest month), BIO8 (mean temperature of wettest quarter), BIO9 (mean temperature of driest quarter), BIO10 (mean temperature of warmest quarter), BIO12 (annual precipitation), BIO13 (precipitation of wettest month), BIO15 (precipitation seasonality), BIO17 (precipitation of driest quarter) and BIO19 (precipitation of coldest quarter).

Ensemble Models (EMs) resulting from the ‘wmean’ algorithm show high values of AUC = 0.936 and TSS = 0.732 for *S*. *perspicillata* and AUC = 0.997 and TSS = 0.976 for *S*. *terdigitata*. Similar scores are also obtained with the ‘median’ algorithm, with AUC = 0.933 and TSS = 0.732 for *S*. *perspicillata*, and AUC = 0.998 and TSS = 0.978 for *S*. *terdigitata*, further confirming the reliability of our models.

The ‘wmean’ maps for the EMs, projected over the current scenario (Supplementary Fig. S2a,b), show a wide continuous suitable area in Apennines for *S*. *perspicillata*, while for *S*. *terdigitata* the predicted suitable area results fragmented in discontinuous patches, some of which outside its known range of occurrence. From the “genus-level” model (AUC = 0.957 and TSS = 0.784), the ‘wmean’ maps show a vast and very-high suitable area occurring throughout the whole peninsula (Supplementary Fig. S2c), except for the Alps and of some parts of the Apulia region and Padano-Venetian plain.

The ‘cv’ maps also report areas with a general low level of uncertainty for both *S*. *perspicillata* (maximum ‘cv’ value = 0.221) and *S*. *terdigitata* (maximum ‘cv’ value = 0.401) (Supplementary Fig. S2d,e). For this latter species, only suitable areas placed outside its current range show the higher levels of ‘cv’ values; a similar response is also observed for the ‘cv’ values for the “genus level” model (Supplementary Fig. S2f).

BIO19 (contribution percentage (cp) = 15.2%), BIO13 (cp = 14.7%), BIO6 (cp = 12.1%) and BIO8 (cp = 11.5%) resulted as the four most contributing variables for *S*. *perspicillata*, while BIO17 (cp = 38.8%), BIO19 cp = 26.8%), BIO2 (cp = 12.5%) and BIO3 (cp = 5.8%) resulted as the four most contributing predictors for *S*. *terdigitata*.

The response plots of the first two most contributing variables for each target species (Supplementary Fig. S3a–d) show that BIO19 plays an important role for *S*. *perspicillata* when exceeding the threshold of 250 mm of precipitation, while the same variable contributes to a lesser extent (range 250 ÷ 320 mm) to raise the predicted suitability for *S*. *terdigitata*. About BIO13, another precipitation-related variable, it directly contributes to raise the predicted suitability for *S*. *perspicillata*, when increasing. BIO17, the first contributing variable for *S*. *terdigitata*, slightly raises the predicted suitability in the range between 50 ÷ 110 mm.

**Figure 2:**
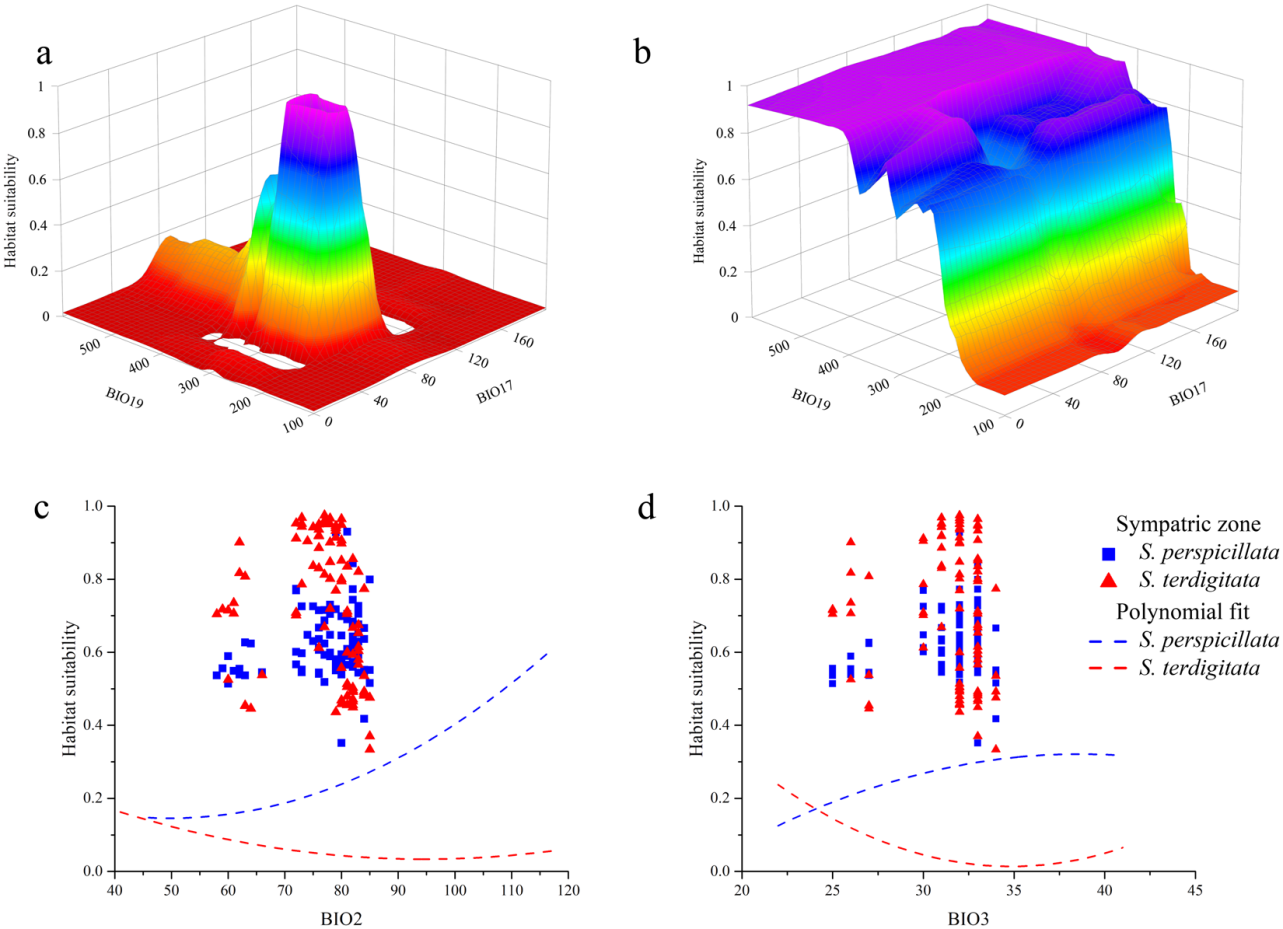
Response plots of climate influence. (**a**) 3-D plot of Habitat suitability as a function of the pairwise interaction between BIO17 (precipitation of the driest quarter) and BIO19 (precipitation of the coldest quarter) for *Salamandrina terdigitata*. (**b**) 3-D plot of Habitat suitability as a function of the pairwise interaction between BIO17 (precipitation of the driest quarter) and BIO19 (precipitation of the coldest quarter) for *Salamandrina perspicillata*. (**c**) Marginal response curves for *S*. *perspicillata* (blue) and *S*. *terdigitata* (red) of BIO2 (mean diurnal range); in blue squares and red triangles, the Habitat suitability as a function of BIO2 sampled in the sympatric zone. (**d**) Marginal response curves for *S*. *perspicillata* (blue) and *S*. *terdigitata* (red) of BIO3 (isothermality); in blue squares and red triangles, the Habitat suitability as a function of BIO3 sampled in the sympatric zone.

Notwithstanding the relatively low contributions for the first two variables in *S*. *terdigitata*, the pairwise interaction between them resulted instead to be very important for predicted suitability (Fig. 2a); on the contrary, no remarkable interaction for the same variable pair is inferred for *S*. *perspicillata* (Fig. 2b), with BIO19 following the same trend of its respective response plot (cf. Supplementary Fig. S3a).

Further, considering the results obtained from the historical “stable areas” and climate heterogeneity for the two target species (see below), response plots of the two variables among all the selected predictors better representing the climatic stability, namely BIO2 and BIO3, were calculated (Fig. 2c,d): an inverse trend is observed, in both variables, for *S*. *perspicillata* and *S*. *terdigitata*. The ‘average’ values of these two variables, when extracted in the predicted binarized sympatric zone, show medium-to-high values of habitat suitability for both species (Fig. 2c,d).

**Figure 3:**
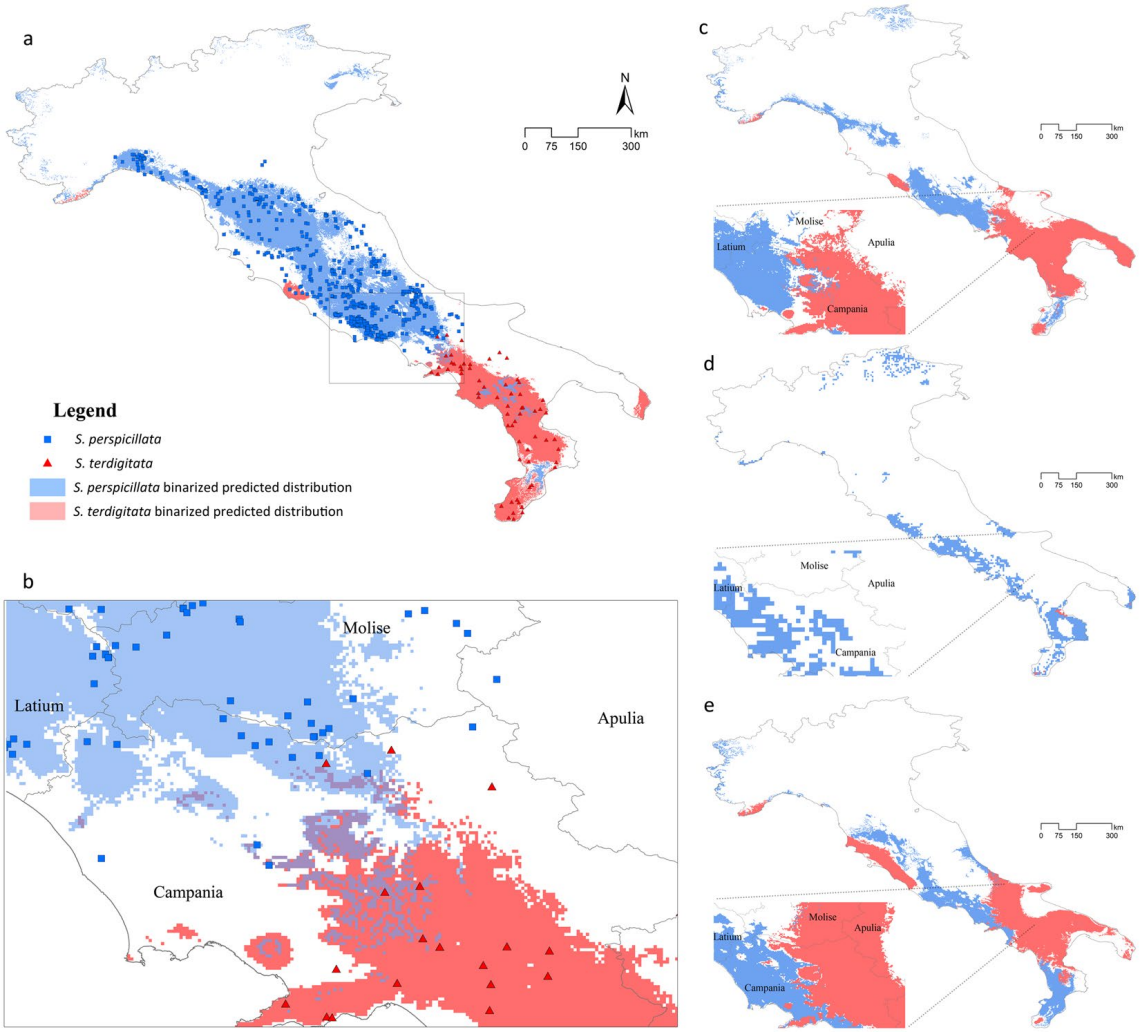
Binarized predictions for target species. (**a**) Binarized predictions for current scenario and presence localities for *Salamandrina perspicillata* (blue) and *S*. *terdigitata* (red). (**b**) Focus over the overlap zone of the binarized current predictions and species’ localities, as in (**a**). (**c**) Binarized predictions for Mid-Holocene scenario, with a focus over the sympatric zone (beside). (**d**) Binarized predictions for Last Glacial Maximum scenario, with a focus over the sympatric zone (beside). (**e**) Binarized predictions for Last Interglacial scenario, with a focus over the sympatric zone (beside).

The TSS-max algorithm resulted in a threshold = 0.533 for *S*. *perspicillata* and TSS-max = 0.432 for *S*. *terdigitata*; these values were used to binarize EMs for both current and past conditions. From the binarized maps reporting the predicted current suitability (Fig. 3a), a continuous and wide Apennine area is predicted for *S*. *perspicillata*, from the northern to the central-lower parts of this mountainous chain; some small and fragmented areas in Lucanian and Calabrian Apennines are predicted as suitable, too. On the other side, binarized areas for *S*. *terdigitata* were predicted for territories actually occupied by the species (lower and southern Apennines), with two other patches (Thyrrenian Tuscany and southern Apulia) predicted as suitable but not currently inhabited by the species. A restricted territory falling in the southern Apennines (sensu Minelli, *et al*.^30^, modified by Biondi, *et al*.^31^), in particular an area between the Sannio-Matese Apennine and the northern portion of the Campanian Apennines hosts both of the binarized areas predicted as suitable for both the target species (Fig. 3b). This sympatric territory is also predicted, even if a western “shift” is observable, in the MOL binarized predicted suitable areas (Fig. 3c), while during the LGM this area (as well as many other territories) were predicted as unsuitable for *S*. *terdigitata* and suitable for *S*. *perspicillata* only (Fig. 3d); the LIG binarized scenario, instead, reports suitable areas for both the two target species, even if an overlap is not observed (Fig. 3e).

### Climate-triggered shifts

**Figure 4:**
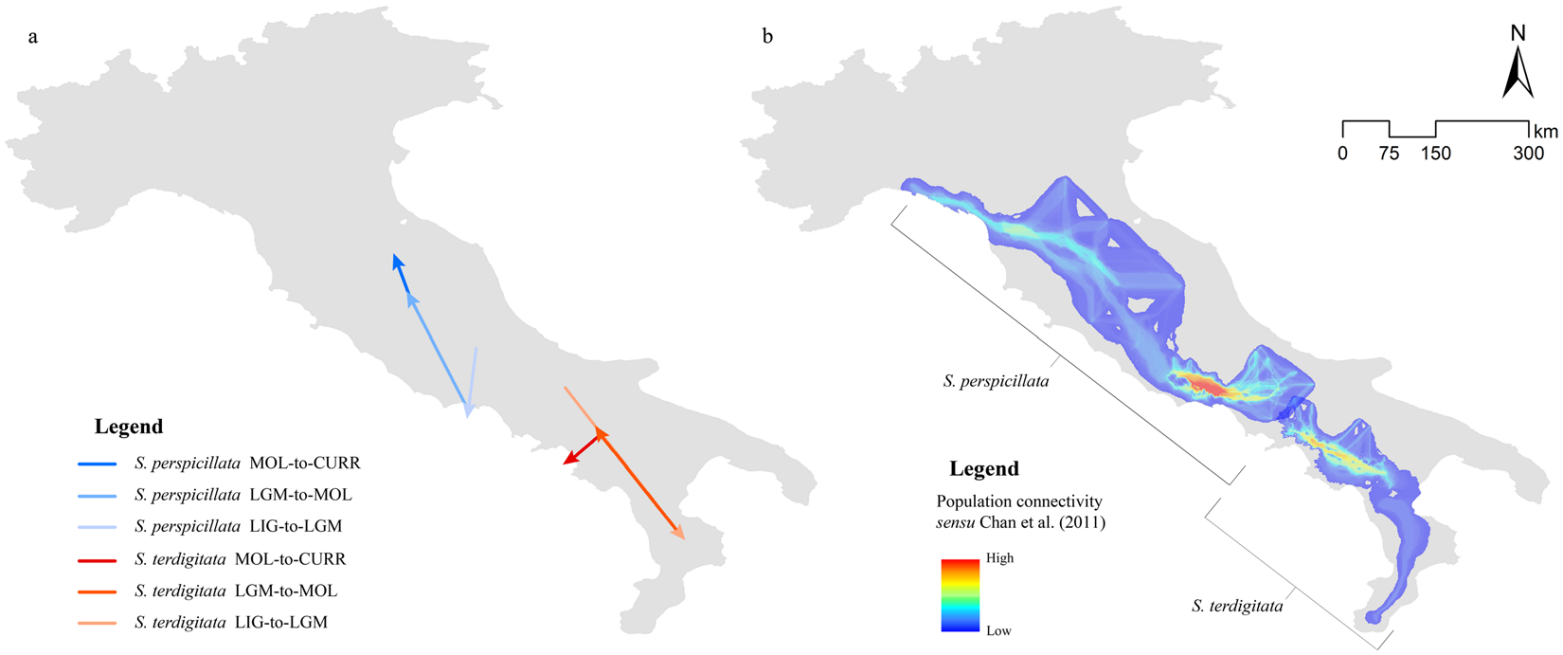
Maps of predicted changes in distribution. (**a**) Shifts of predicted binarized areas’ (*Salamandrina perspicillata*: blue shades; *S*. *terdigitata*: red shades) centroids between each different time-frame considered. (**b**) Haplotype dispersal network (*sensu* Chan, *et al*.^74^) calculated over the predicted Mid-Holocene climatic scenario for the two target species.

The results from the ‘Centroid changes’ tool used over these binarized predictions reveal a general southern shift during the LIG-to-LGM period for both the target species, and a subsequent movement northward during the LGM-to-MOL period; the MOL-to-Current results reveal a further northward expansion for *S*. *perspicillata*, and a western shift for *S*. *terdigitata* (Fig. 4a). Moreover, the Least-Cost Pathways calculated through the MOL predicted scenarios and the haplotype network for both the target species, reveal a higher use of the Volsci chain in *S*. *perspicillata* and the southern Apennines in *S*. *terdigitata* for migrations. Some connectivity is also predicted in northern Apennines and in central Apennines (towards the eastern side), while small connectivity is calculated for the Calabrian Apennines; a contact zone between corridors calculated for the two target species is predicted in the Sannio-Matese Apennine.

### Stable areas

**Figure 5:**
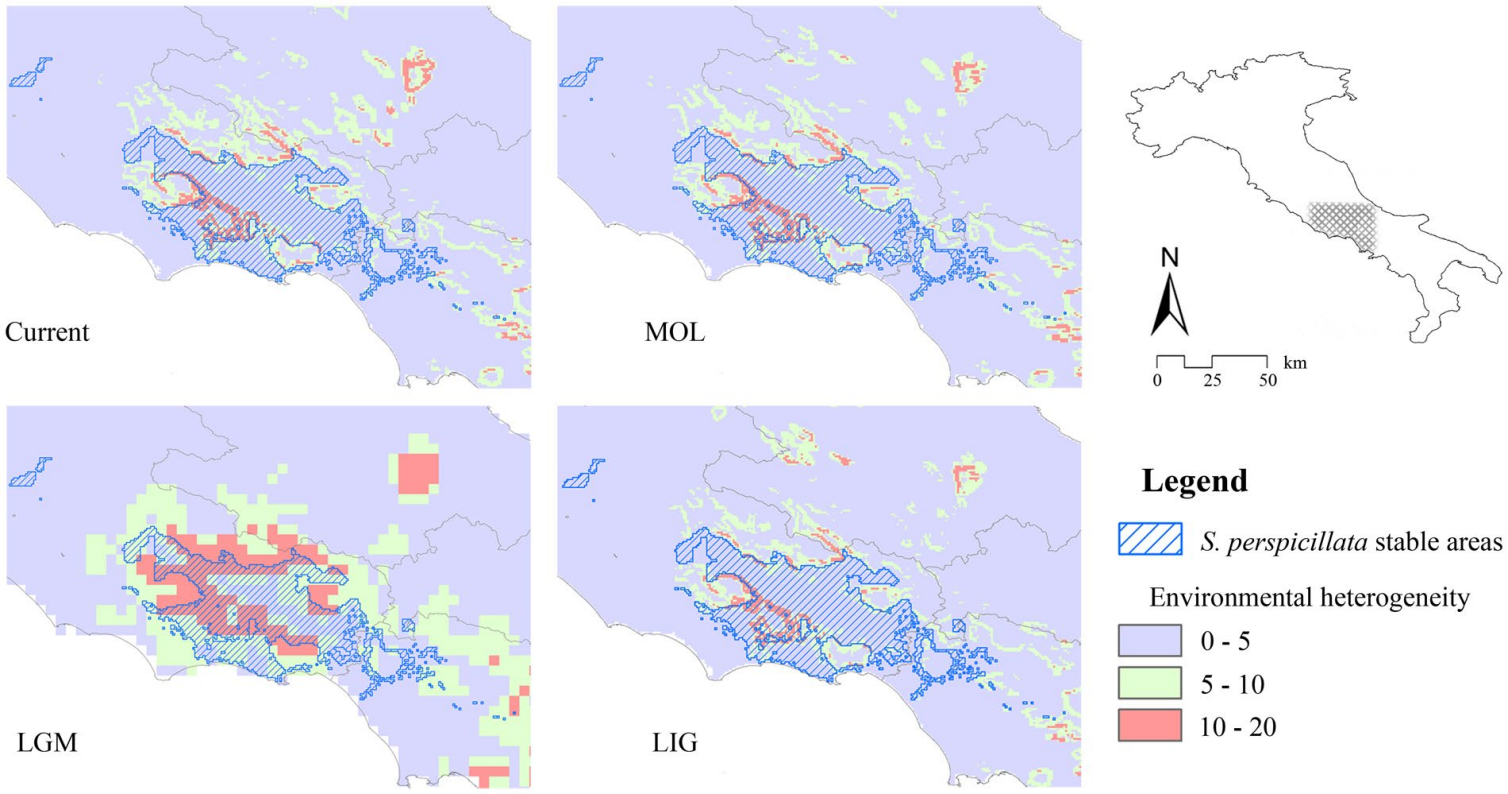
Maps of environmental heterogeneity and stable areas. Areas predicted as stable (suitable for all the time-frames considered) for *Salamandrina perspicillata* (blue hatch), overlaid to the maps of environmental heterogeneity calculated over the climatic conditions for each time-frame considered.

**Figure 6:**
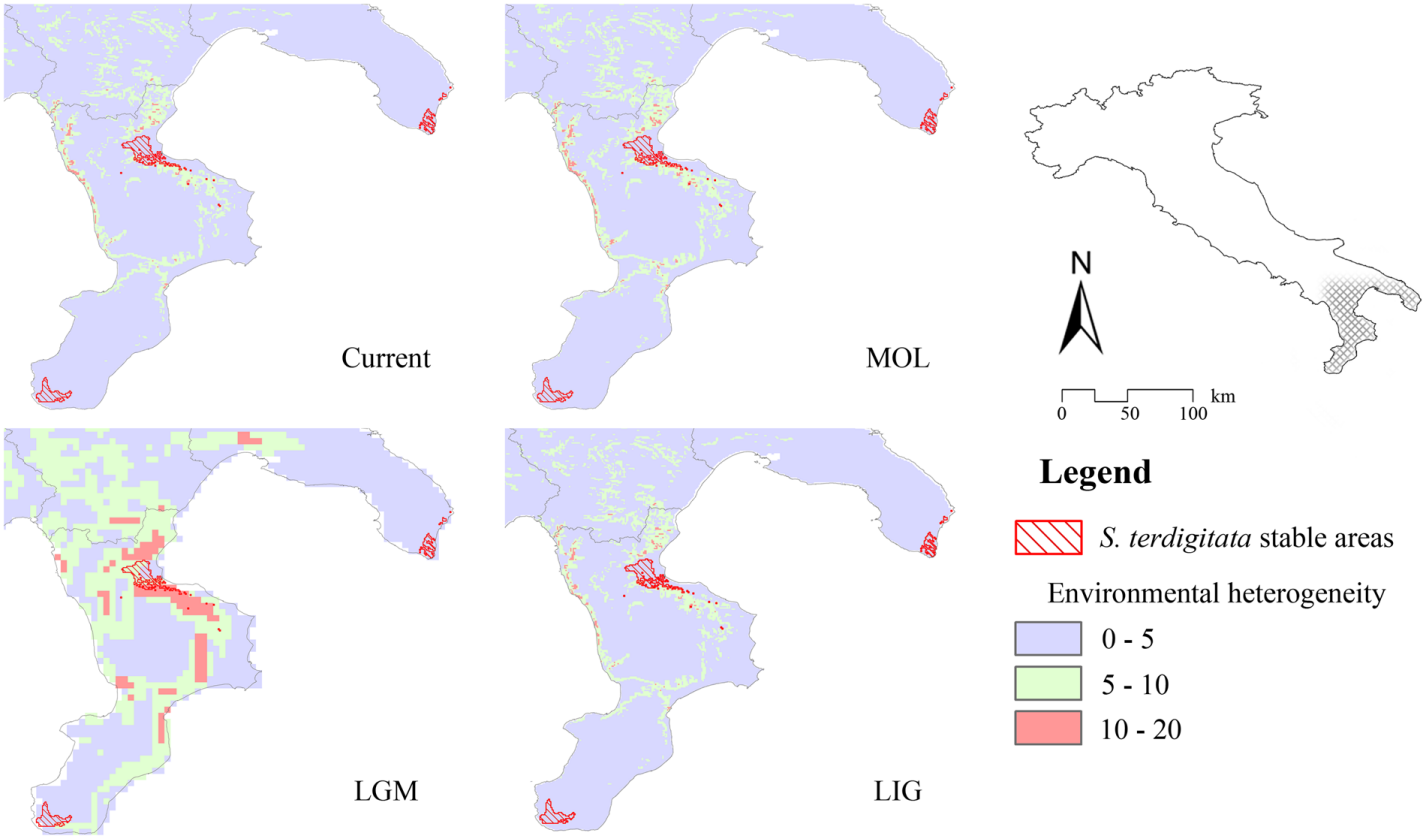
Maps of environmental heterogeneity and stable areas. Areas predicted as stable (suitable for all the time-frames considered) for *Salamandrina terdigitata* (red hatch), overlaid to the maps of environmental heterogeneity calculated over the climatic conditions for each time-frame considered.

The intersection among all binarized predicted suitability for all the time frames considered show that, for *S*. *perspicillata*, an area located in Latial Apennines, including Lepini, Ausoni and Aurunci mountains (all belonging to the Volsci chain), remained stable over time (Fig. 5). On the other side, an area falling in a mountain-valley system in Calabria, which currently hosts part of the Pollino National Park, and another area located in the southern part of Calabria, are the predicted stable areas for *S*. *terdigitata* (Fig. 6); in these territories, the climatic heterogeneity remained rather low from LIG to Current time-frames, while the stable area for *S*. *perspicillata* resulted in higher climatic heterogeneity with respect to its congeneric. Also, a small area in Salento (southern part of Apulia) in predicted to be stable over all the time-frames considered resulted for *S*. *terdigitata*, even though this area is not currently inhabited by the species.

### Haplotype network

**Figure 7:**
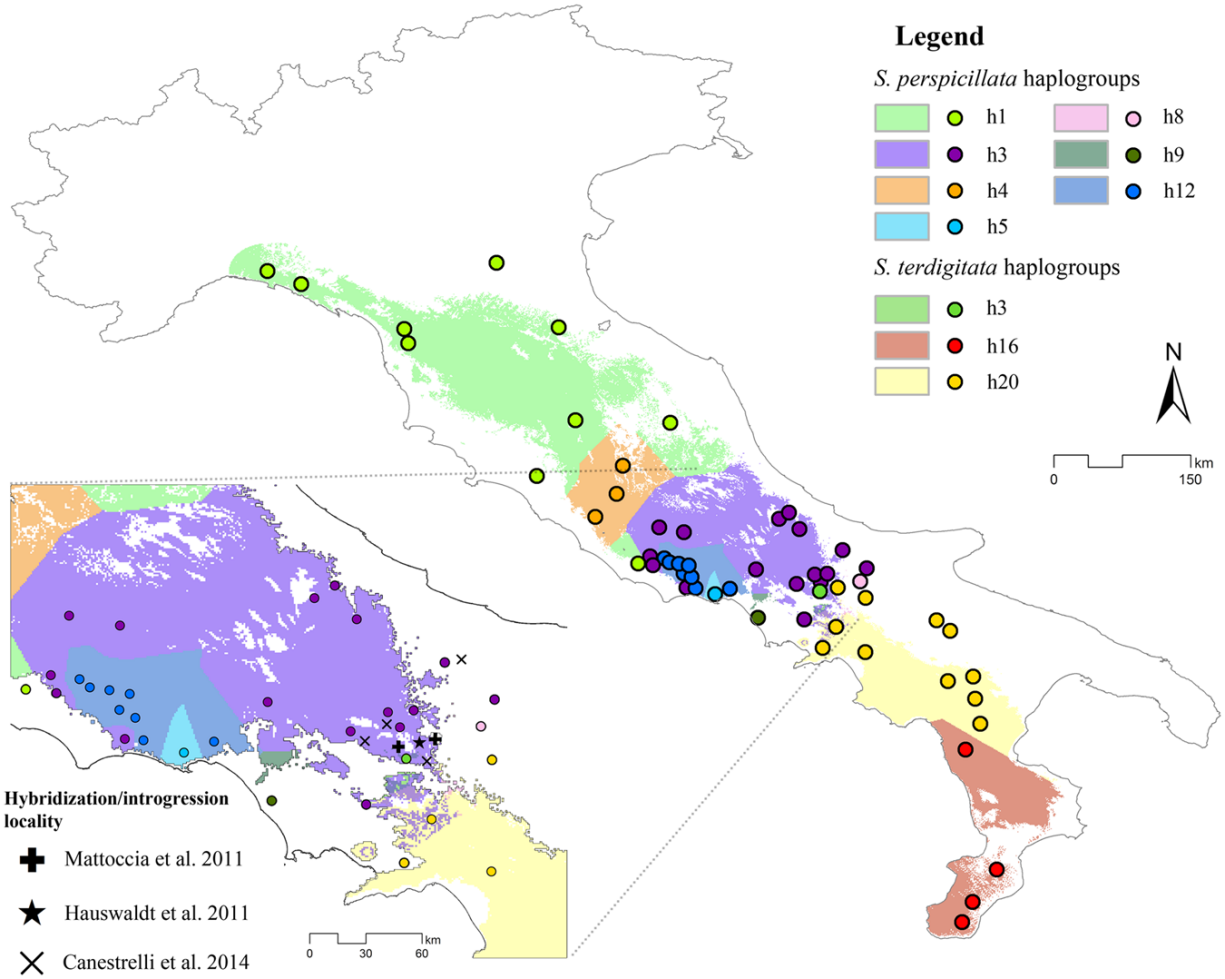
Voronoi polygons and haplotype network. Binarized suitable areas for the tow target species split by Voronoi polygons calculated through the haplotype network of Mattoccia, *et al*.^15^. Besides, a focus on the contact zone between Salamandrina perspicillata and S. terdigitata, also highlighting the hybridization localities from^15,23,24^.

As regards the haplotype network^15,23^, the ‘Split binary SDM by input clade relationship’ tool revealed many patches of binarized suitable area for *S*. *perspicillata*, with the higher density of Voronoi polygons (corresponding to higher haplotype diversity) in the anti-Apennines, while two vast polygons divide the southern territories of *S*. *terdigitata*. The overlap of the binarized predictions (see above) is also observed in the obtained Voronoi polygons’ areas belonging the h3, h9, h8 and h3, h20 haplotypes (cf. Mattoccia, *et al*.^15^) for *S*. *perspicillata* and *S*. *terdigitata*, respectively (Fig. 7).

## Discussion

The distribution obtained from the Ensemble Models and GIS analyses show a high conformity among each other: the Minimum Convex Polygons (MCPs) (Fig. 1) match the shape of the predicted suitability (Fig. 3a), with some minor exceptions, for both the two target species. In the Central-Southern Apennines, the binarized Ensemble Models for both species show an area of overlap (Fig. 3b). Other minor patches falling outside of the current range of both the target species analyzed are predicted from the Ensemble Models (Fig. 3a), such as the small areas in Campania (Central-Southern Italy) and central Calabria (Southern-Western Italy) for *S*. *perspicillata* and the northern part of Latium (Central-Western Italy) and south Apulia (Southern-Eastern Italy) for *S*. *terdigitata*. Variables’ contributions generally show a trend in which *S*. *terdigitata* seem to require more stable climatic conditions than *S*. *perspicillata*; this is observable in the interaction between the Precipitation of Driest (BIO17) and Coldest (BIO19) Quarter, in which the predicted suitability sharply raise within a restricted range of both (Fig. 2a), and in the contrasting trends of the Mean Diurnal Range (BIO2) and Isothermality (BIO3) curves for the two species (Fig. 2c,d). Also, the ‘average’ ranges of these two variables observed in the predicted sympatric zone result in medium-to-high habitat suitability for both species, suggesting that intermediate conditions of climate stability may promote their co-occurrence. In addition, merging the two target species’ datasets (i.e. calibrating through climatic requirements of both target species), the genus-level Ensemble Model confirm the climatic separation between the two species, considering that a “flat”, poorly-informed response is returned. For instance, no discrimination is observable among climates of coastal, hilly and mountainous areas, with a very high habitat suitability in most of the Italian peninsula (Supplementary Fig. S2c). A significant separation of the two species is also confirmed by the Discriminant Analysis based on the twelve climatic variables considered, with a total percentage of 96.2% corrected attributions (Supplementary Fig. 4).

The changes in species’ distributions as the consequence of the climate influences during the Last Glacial Maximum and following periods^2,9,32,33^ can be observed through the shifts calculated between each time frame we considered and centroids of areas predicted as suitable, for both the two target species. In fact, a southward shift is calculated both for *S*. *perspicillata* and *S*. *terdigitata*, with the former taking refuge in Southern Latium and the latter taking refuge in Calabria, during the LIG to LGM transition (Fig. 4a).

The predicted northwards expansions after the LGM period (LGM-to-MOL and MOL-to-CURR) are, for both the target species analyzed (Fig. 4a), in agreement with what hypothesized by the models of the European postglacial expansion^2^.

In this scenario, the postglacial expansion and the secondary contact inferred through genetic data^15,24^ are also confirmed by our Least Cost Pathways (Fig. 4b): the northwards expansion of *S*. *perspicillata* reveals Apennine corridors used for the recolonization, which possibly favored the segregation of two widespread haplotypes (see below), while the Volsci area (Southern Latium) was more frequently used for the colonization of Central Apennines, in which populations show higher genetic diversity^15,23^.

Moreover, *S*. *terdigitata* re-colonized Campanian Apennines by using the re-established higher climatic suitability of the MOL period, thus creating an area of sympatry as hypothesized by Mattoccia, *et al*.^15^ and Canestrelli, *et al*.^24^ right for the post-glacial conditions, in which the “secondary contact” could have formed. The low use of corridors in the southern part of *S*. *terdigitata* range, Calabrian Apennines (Fig. 4b), matches with the genetic data of Hauswaldt, *et al*.^25^, who estimated no contacts among populations in the post-glacial period.

The influence of climate is also clear when analyzing the predicted areas which remained stable over all the time-frames considered: *S*. *perspicillata* has been occupying (and continues to do) a single large area in southern Latium, which is characterized by a high climate heterogeneity at least since the LIG period (Fig. 5). This result agrees with the genetic data and the related areas outlined by Mattoccia, *et al*.^15^ and in Hauswaldt, *et al*.^25^, where a single glacial refugium is identified in southern Latium – northern Campania, in the Volsci Mountain Chain. Further, some genetic data showed higher diversity at about 80 km distance from the Volsci Chain^25^, thus leading to the hypothesis of an alternative refugia, which is predicted by our analyses to be suitable only in the MOL and Current scenario (Fig. 3a,c).

On the contrary, *S*. *terdigitata* is predicted to have its main stable areas in two separate territories, located in the Sibari plain (northern Calabria, at the foot of Pollino Massif) and at the tip of Calabria (at the foot of Aspromonte Massif) (Fig. 6). An area falling within the Salento peninsula is also predicted to be suitable for *S*. *terdigitata* and connected to the rest of the predicted range only during the MOL period (Fig. 3c), even though this area has never been occupied from this species. Again, our results fully agree with the latest genetic scenario, in which multiple areas are predicted to have acted as refugia during the LGM^25^. Recently, *S*. *terdigitata* was found in the north-western side of Apulia^34^, in a rather disjunct locality with respect to the others occurrences of this species. Indeed, more research about the autoecology of this species is needed, so as to better characterize its ecological niche and understand its current (and possible past) distribution.

Finally, considering the haplotype network proposed by Mattoccia, *et al*.^15^, we can observe that the most ancestral ones are distributed in two main areas. For *S*. *perspicillata*, a large area in central Italy, as defined through the Voronoi polygons, harbors the “h3” haplotype, while the “h16” Voronoi polygon covers the whole predicted suitable area of Calabria (Fig. 7) for *S*. *terdigitata*. These findings suggest that suitable climatic conditions in these areas shaped the populations’ genetics, with the ancestral haplotypes rooted in some territory since LGM, recolonizing the areas outside the “stable zones” (cf. Figs 5 and 6) in a certain range. The area in the southern Apennines hosts many different haplotypes, in which the corresponding Voronoi polygons overlap each other in a complex and genetic-dense territorial asset; this area is identified as the “secondary contact” zone, and, in fact, hosts the hybridization localities reported by Mattoccia, *et al*.^15^ and Hauswaldt, *et al*.^23^ (Fig. 7). To disentangle the role of climate in species’ divergence, introgression and hybridization phenomena, more sampling effort should be applied to the overlap zone, predicted over climatic conditions, that we map in this paper. In fact, the sampling performed until now did not possibly cover the core of the hybrid zone analyzed^24^, which is predicted by our models to occur also at lower latitudes.

Our spatial patterns can be compared with the ones found in other researches on Caudata: a cycle of contraction/recolonization was observed, for instance, in Dinarides and Alps for *Salamandra atra* at a subspecies level^35^, and in peninsular Italy, where a similar pattern was recently observed in the two parapatric newts *Lissotriton vulgaris meridionalis* and *L*. *italicus*^36^.

In conclusion, our analyses fully meet the hypotheses of climate shaping the distribution of European fauna before, during and after the Last Glacial stage; the southward migrations and the subsequent northward re-colonizations, hypothesized through genetic data, are confirmed by our direct analyses of climatic influences. Also, issues related to genetic evidences, such as the genetic richness within glacial refugia, the post-glacial recolonization and the establishment of a secondary contact area in the endemic *Salamandrina* genus are observed and mapped in specific zones by coupling Ensemble Modelling techniques and GIS-based post-modelling analyses, highlighting the importance of genetic and historical data analysis through spatial processes. Moreover, the effect that past climate exerted over *Salamandrina* species should be considered for present-day management purposes: in fact, the ongoing climate change could directly influence the two species, leading to conservation issues.

## Methods

### Target species and database

Two amphibians belonging to the Italian endemic genus *Salamandrina*, *S*. *perspicillata* (Northern spectacled salamander) and *S*. *terdigitata* (Southern spectacled salamander), were considered as target species in our analyses. *Salamandrina perspicillata* and *S*. *terdigitata* are both forest-dwellers amphibians, linked to the forest’s shade and moisture conditions, living mainly along the Apennine chain; the life cycle shows a long terrestrial phase, which is interrupted only for the reproductive activities; the mating and egg deposition are, in fact, the only phases in which the use of streams is observed^37^. These species are distributed over the peninsular Italy (Fig. 1), from the Ligurian to the Calabrian Apennines, showing a parapatric distribution^15^ and, at least, seven syntopic localities^15,23,24^.

For the aims of this paper, we generated a GPS-precision occurrence records database, integrating literature search and field observations, for both the target species (Supplementary Table S5). To avoid spatial autocorrelation among records, a spatial thinning process was performed through the spThin package^38^ in R^39^; a further validation of the occurrence dataset obtained after the thinning process was performed calculating the Morans’ I in ArcMap 10.0^40^. A database obtained merging the two species’ post-thinning datasets into a “genus-level” one was also generated.

### Model building and calibration

In order to obtain SDMs for the two target species, nineteen bioclimatic variables were downloaded from the Worldclim.org repository (ver.1.4)^41^ for current (CURR), Mid-Holocene (MOL, ~6000 years ago), Last Glacial Maximum (LGM, ~22000 years ago) and Last inter-glacial (LIG, ~120000–140000 years ago) conditions, at 30 arc-seconds spatial resolution, with an exception for LGM, available only at 2.5 min resolution. For paleoclimatic conditions, we selected two shared Global Climate Models (GMCs), the CCSM4^42^ and the MIROC-ESM^43^, available both for MOL and LGM; for the LIG paleoclimate, the only available GCM is the one from Otto-Bliesner, *et al*.^44^. A correlation matrix was computed in ArcMap 10.0 among all the nineteen candidate predictors (Supplementary File S6), to select variables’ pairs showing a Pearson’s |r| > 0.85^45^, and subsequently exclude the ones, in each pair, having less influence, considering the bibliographic information about the target species’ ecological requirements^37,46^. The SDMs for the two target species and for all the different time-frames were built through the implementation of the ‘biomod2’ package^47^ in R environment^39^. Species Distribution Models and cartographic tools are more and more used in recent years to interpret biogeographic processes^36,48,49^, conservation and biodiversity issues^50–53^, giving interesting results when combined^54–58^. This package permits to obtain Ensemble Models, a powerful technique which proportionally combines models obtained from different processes into one single prediction. The single modelling techniques used to calibrate the model on the current climatic conditions were Generalized Linear Models (GLMs), Multivariate Additive Regression Splines (MARS), Gradient Boosting Models (GBM, often named as BRTs) and Maxent; these techniques were selected to analyze responses from different approaches (from the linear regressions to the machine learning techniques); the details about each model parametrization are reported in Supplementary File S7. Ten sets of 1000 pseudo-absences each were generated through the surface range-envelope algorithm (quantile set at 0.05), obtaining pseudo-absence points falling outside the 95^th^ quantile of the linear environmental envelope built on presence points, a technique used when dealing with potentially incomplete species’ distributions^59,60^ which also lowers the commission error^61^. The ‘BIOMOD_Modeling’ function was finally used to calibrate the models with all these settings.

### Model evaluation and ensemble forecast

The models obtained were evaluated through the 20% of the initial dataset (the 80% of the dataset is used to calibrate the initial model) and 5 evaluation runs, for a total of 200 models evaluated (4 modelling techniques × 10 pseudo-absence sets × 5 evaluation runs). The discrimination performance of each model was assessed through two different metrics, the Area Under the Curve (AUC)^62^ of the receiver operator characteristic curve and the True Skill Statistic (TSS)^63^.

All models selected for the Ensemble modelling process exceeded the thresholds of TSS > 0.8 and AUC > 0.7, as a good trade-off between the AUC, a good performance statistics which may sometimes give high scores to overfitted models^59,64^ (a possible condition when using GLMs, as is our case), and the more “independent” TSS^63^. The ‘wmean’ (weighted mean of probabilities, which averages each model based on the respective AUC and TSS scores), the ‘median’ (median of values, which is less sensitive to outliers than the ‘wmean’, as reported in Thuiller, *et al*.^47^) and ‘cv’ (coefficient of variation, which spatially highlights possible conflicts among models used to build the EM) algorithms^47^ were used to build the Ensemble Model through the ‘BIOMOD_EnsembleModeling’ function. Also, predictors’ contributions were assessed and plots of the most contributing variables were obtained through the ‘response.plot2’ function^65^, to investigate the target species’ response to bioclimatic conditions, also calculating possible pairwise interactions; the EM was then projected to the different time-frames through the ‘BIOMOD_EnsembleForecasting’ function. Considering that when projecting to different time-frames (or different territories) with respect to the calibrated models, model extrapolation may occur (i.e. the environmental conditions of a projected scenario differ from the ones used to calibrate the model)^66^, the Multivariate Environmental Surface Similarity (MESS)^67^ was assessed through the ‘mess’ function in the ‘dismo’ package in R^68^. Subsequently, the computed model extrapolation was further processed through the Multivariate Environmental Dissimilarity Index (MEDI)^36^, a procedure which proportionally down-weights extrapolation of each EM built with different GCM when calculating an “average” model for a specific time-frame, as in this case. Finally, a TSS-max threshold was computed through the ‘ecospat’ R package^69^ to obtain binarized models; this procedure, considering that presence-background models were built, is considered reliable for these kind of models, as reported in Liu, *et al*.^70^.

### Post-modelling and GIS analyses

The MEDI-processed Ensemble Models were binarized through the TSS-max threshold by using the ‘Reclassify’ tool in ArcMap 10.0^40^. The shifts of binarized centroids obtained for each time-frame and species were calculated through the ‘Centroid changes’ in the ‘SDMtoolbox 2.1’^71^. In the same toolbox, a Principal Component Analysis (‘Principal Component Analysis’ tool) was performed over the selected predictors, which was subsequently used to assess the environmental heterogeneity (‘Heterogeneity Calculation’ tool) during all the time-frames considered.

To compare predicted past and current models obtained with the genetic network information available for the two target species in Hauswaldt, *et al*.^23^ and in Mattoccia, *et al*.^15^, the ‘Split binary SDM by input clade relationship’ tool in ‘SDMtoolbox 2.1’^71^ was used. This tool permits to classify the binarized predictions obtained for the current EMs’ scenarios into areas belonging to a certain clade, separating geographic space through Voronoi polygons^72,73^. Minimum Convex Polygons were built with the ‘Minimum Bounding Geometry’ tool (setting the ‘Convex Hull’ as bounding geometry) in ArcMap 10.0^40^ and subsequently clipped to the extent of the study area. Also, the ‘Least Cost Pathways’ tool (*sensu* Chan, *et al*.^74^) was used to calculate the possible corridors used after the LGM, as suggested by the findings of Mattoccia, *et al*.^15^, thus for the MOL climatic conditions, for both the target species.

The ‘Extract multi values to points’ tool in ArcMap 10.0^40^ was used to gather climatic conditions from Worldclim bioclimatic variables in each target species’ occurrence locality. A Linear Discriminant Analysis was performed with these data to derive functions discriminating three groups, namely *S*. *perpicillata*, *S*. *terdigitata* and *S*. *perspicillata* x *terdigitata*, with this latter indicating the syntopic localities^15,23,24^. No data standardization or normalization was performed for these variables.

All supporting layers (shapefiles, rasters) used to perform the over-cited GIS analyses were obtained through geoprocesses in ArcMap 10.0^40^.

## Electronic supplementary material

Supplementary Files

Supplementary Table S5
