# Supplementary material for: Evidences for a shared history for spectacled salamanders, haplotypes and climate
Source: Sci Rep. 2018 Nov 7;8:16507. doi: 10.1038/s41598-018-34854-1 (PMC6220306; doi:10.1038/s41598-018-34854-1)
Supplement: Supplementary file 1 — Supplementary Files [file 41598_2018_34854_MOESM1_ESM.docx]

**Evidences for a shared history for spectacled salamanders, haplotypes and climate**

**Mattia Iannella^1,^*, Paola D’Alessandro^1^, and Maurizio Biondi^1^**

^1^ University of L’Aquila, Department of Health, Life, and Environmental Sciences, L’Aquila, 67100, Italy

*mattia.iannella@univaq.it

**Supplementary Files**

**Supplementary Table S1.** Table reporting the correlation matrix (Pearson’s r) calculated for the nineteen bioclimatic candidate predictors. Variable pairs exceeding the threshold value (set to |r| > 0.85) are highlighted in yellow.

|  | BIO1 | BIO2 | BIO3 | BIO4 | BIO5 | BIO6 | BIO7 | BIO8 | BIO9 | BIO10 | BIO11 | BIO12 | BIO13 | BIO14 | BIO15 | BIO16 | BIO17 | BIO18 | BIO19 |
| --- | --- | --- | --- | --- | --- | --- | --- | --- | --- | --- | --- | --- | --- | --- | --- | --- | --- | --- | --- |
| BIO1 | 1.00 |  |  |  |  |  |  |  |  |  |  |  |  |  |  |  |  |  |  |
| BIO2 | -0.32 | 1.00 |  |  |  |  |  |  |  |  |  |  |  |  |  |  |  |  |  |
| BIO3 | -0.56 | 0.44 | 1.00 |  |  |  |  |  |  |  |  |  |  |  |  |  |  |  |  |
| BIO4 | 0.27 | -0.44 | -0.54 | 1.00 |  |  |  |  |  |  |  |  |  |  |  |  |  |  |  |
| BIO5 | 0.08 | -0.32 | -0.57 | 0.66 | 1.00 |  |  |  |  |  |  |  |  |  |  |  |  |  |  |
| BIO6 | -0.47 | 0.05 | -0.18 | 0.51 | 0.70 | 1.00 |  |  |  |  |  |  |  |  |  |  |  |  |  |
| BIO7 | 0.84 | -0.15 | -0.79 | 0.34 | 0.26 | -0.19 | 1.00 |  |  |  |  |  |  |  |  |  |  |  |  |
| BIO8 | 0.22 | -0.10 | 0.14 | 0.16 | -0.59 | -0.37 | 0.03 | 1.00 |  |  |  |  |  |  |  |  |  |  |  |
| BIO9 | 0.50 | 0.43 | 0.25 | -0.28 | -0.44 | -0.58 | 0.39 | 0.26 | 1.00 |  |  |  |  |  |  |  |  |  |  |
| BIO10 | 0.02 | -0.30 | -0.54 | 0.63 | 0.99 | 0.72 | 0.22 | -0.60 | -0.47 | 1.00 |  |  |  |  |  |  |  |  |  |
| BIO11 | -0.64 | 0.50 | 0.97 | -0.53 | -0.50 | -0.09 | -0.82 | 0.05 | 0.18 | -0.47 | 1.00 |  |  |  |  |  |  |  |  |
| BIO12 | 0.28 | -0.42 | -0.52 | 0.99 | 0.64 | 0.50 | 0.33 | 0.19 | -0.26 | 0.61 | -0.51 | 1.00 |  |  |  |  |  |  |  |
| BIO13 | 0.81 | -0.35 | -0.72 | 0.35 | 0.26 | -0.20 | 0.85 | 0.07 | 0.26 | 0.22 | -0.82 | 0.34 | 1.00 |  |  |  |  |  |  |
| BIO14 | 0.25 | -0.46 | -0.60 | 0.94 | 0.86 | 0.60 | 0.34 | -0.16 | -0.36 | 0.83 | -0.57 | 0.92 | 0.36 | 1.00 |  |  |  |  |  |
| BIO15 | 0.32 | 0.16 | 0.40 | -0.29 | -0.47 | -0.52 | 0.06 | 0.35 | 0.70 | -0.49 | 0.23 | -0.27 | 0.32 | -0.39 | 1.00 |  |  |  |  |
| BIO16 | 0.86 | -0.14 | -0.75 | 0.31 | 0.21 | -0.24 | 1.00 | 0.08 | 0.45 | 0.16 | -0.79 | 0.31 | 0.86 | 0.31 | 0.14 | 1.00 |  |  |  |
| BIO17 | 0.82 | 0.13 | -0.46 | 0.12 | 0.00 | -0.37 | 0.90 | 0.14 | 0.74 | -0.05 | -0.50 | 0.13 | 0.71 | 0.09 | 0.35 | 0.92 | 1.00 |  |  |
| BIO18 | 0.54 | 0.45 | 0.10 | -0.20 | -0.34 | -0.51 | 0.52 | 0.20 | 0.97 | -0.37 | 0.06 | -0.19 | 0.29 | -0.27 | 0.55 | 0.56 | 0.83 | 1.00 |  |
| BIO19 | 0.58 | -0.57 | -0.62 | 0.85 | 0.62 | 0.13 | 0.54 | 0.07 | -0.09 | 0.57 | -0.63 | 0.84 | 0.55 | 0.85 | -0.17 | 0.52 | 0.34 | -0.01 | 1.00 |

**Supplementary Figure S2.** (**a**) Habitat suitability map resulting from the Ensemble Model (‘wmean’ algorithm) calculated for current climatic conditions for *S. perspicillata*; (**b**) Habitat suitability map resulting from the Ensemble Model (‘wmean’ algorithm) calculated for current climatic conditions for *S. terdigitata*; (**c**) Habitat suitability map resulting from the Ensemble Model (‘wmean’ algorithm) calculated for current climatic conditions for the whole genus *Salamandrina*; (**d**) Coefficient of variation map resulting from the Ensemble Model (‘cv’ algorithm) calculated for current climatic conditions for *S. perspicillata*; (**e**) Coefficient of variation map resulting from the Ensemble Model (‘cv’ algorithm) calculated for current climatic conditions for *S. terdigitata*; (**f**) Coefficient of variation map resulting from the Ensemble Model (‘cv’ algorithm) calculated for current climatic conditions for the whole genus *Salamandrina*.

**
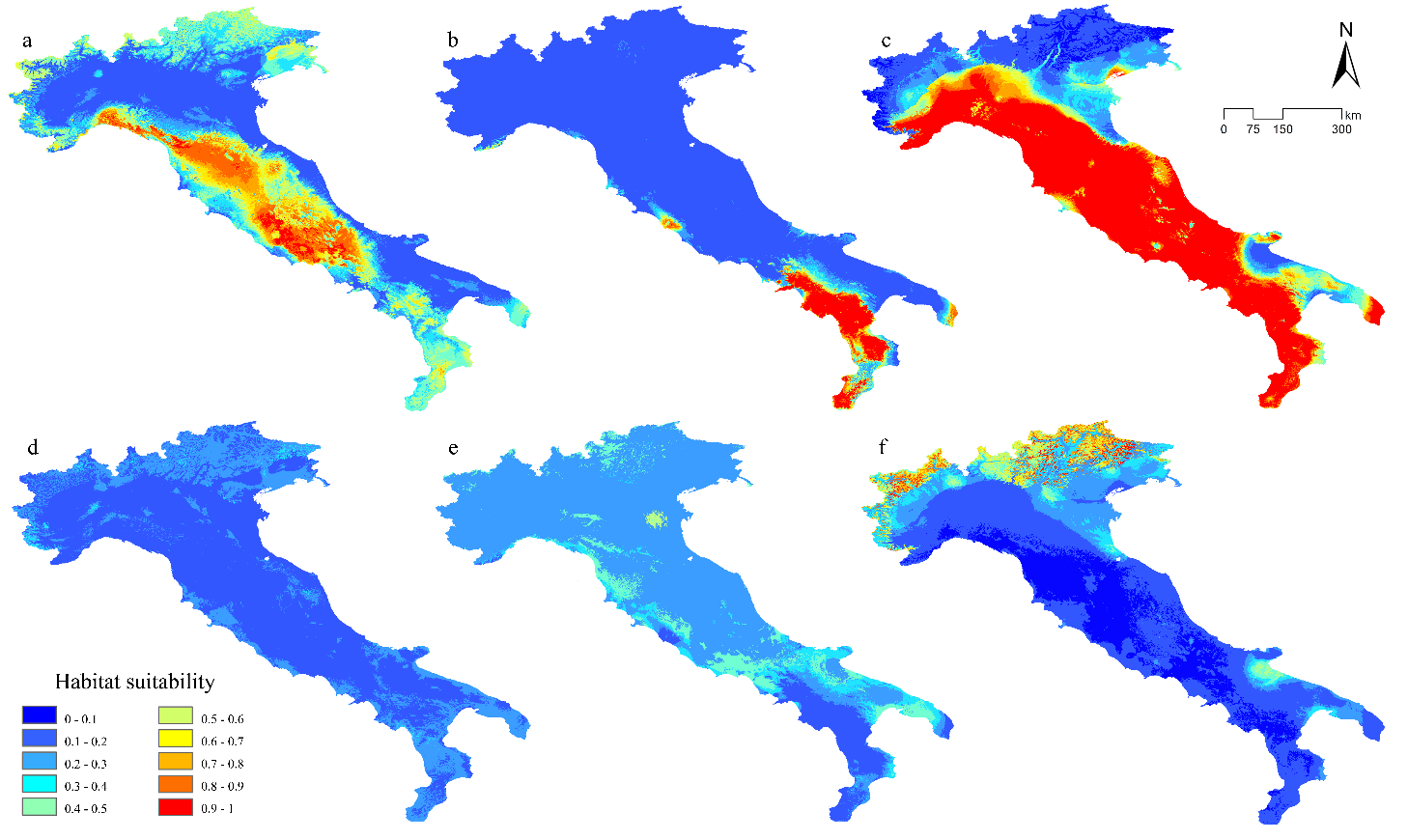
**

**Supplementary Figure S3. (a)** Response curve for the precipitation of the coldest quarter (BIO19) for *S. perspicillata*; **(b)** Response curve for the precipitation of the wettest month (BIO13) for *S. perspicillata*; **(c)** Response curve for the precipitation of the driest quarter (BIO17) for *S. terdigitata*; **(d)** Response curve for the precipitation of the coldest quarter (BIO19) for *S. terdigitata.*

**
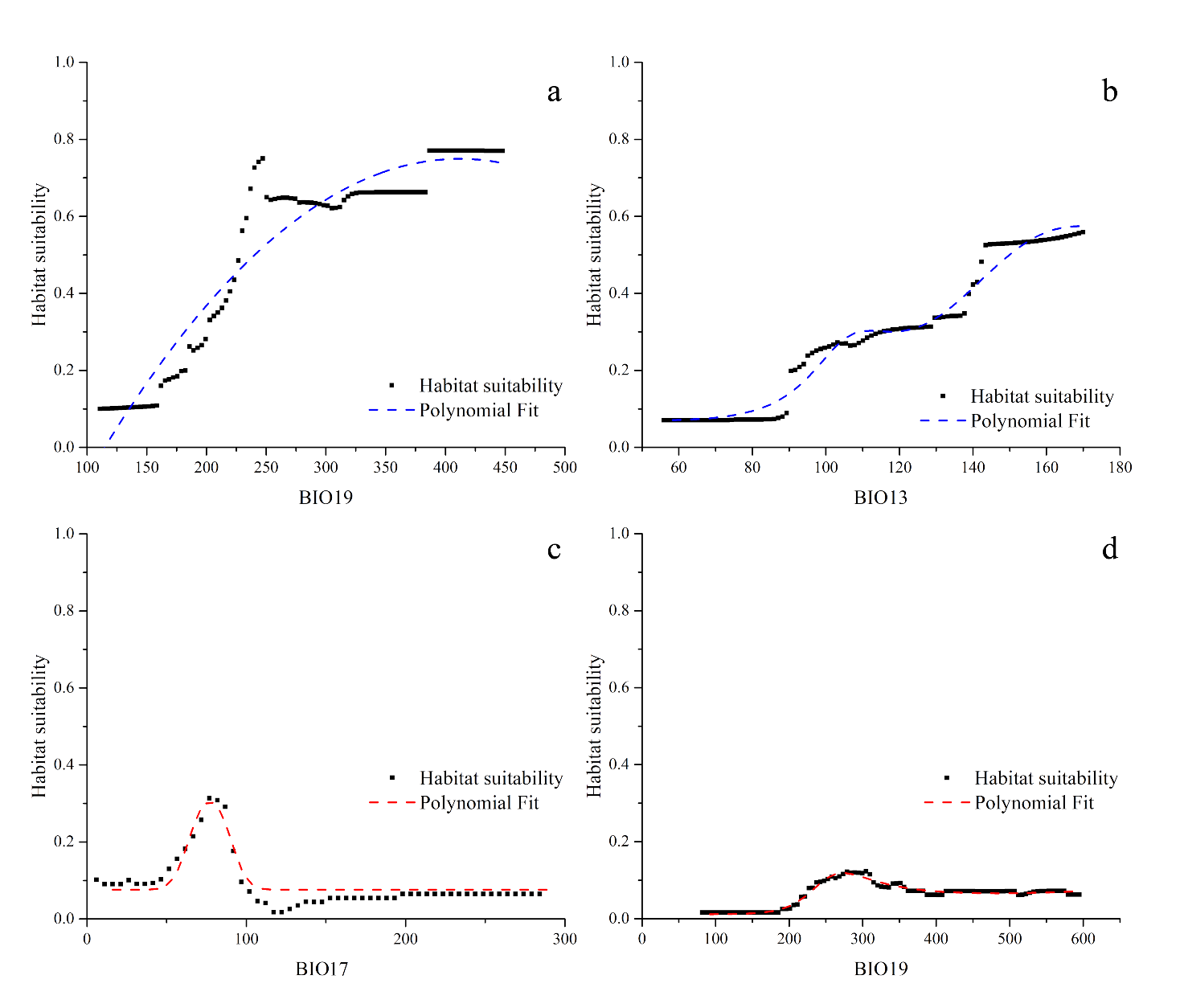
**

**Supplementary Figure S4.** Scatterplot of the two Canonical Variables (CV1 and CV2) resulting from the Linear Discriminant Analysis performed over the twelve bioclimatic variables selected sampled in the occurrence localities for *S. perspicillata* (blue squares), *S. terdigitata* (red triangles) and syntopic localities (*S. perspicillata* x *terdigitata*, green circles). Below, a table reporting the classification count table for the three groups.
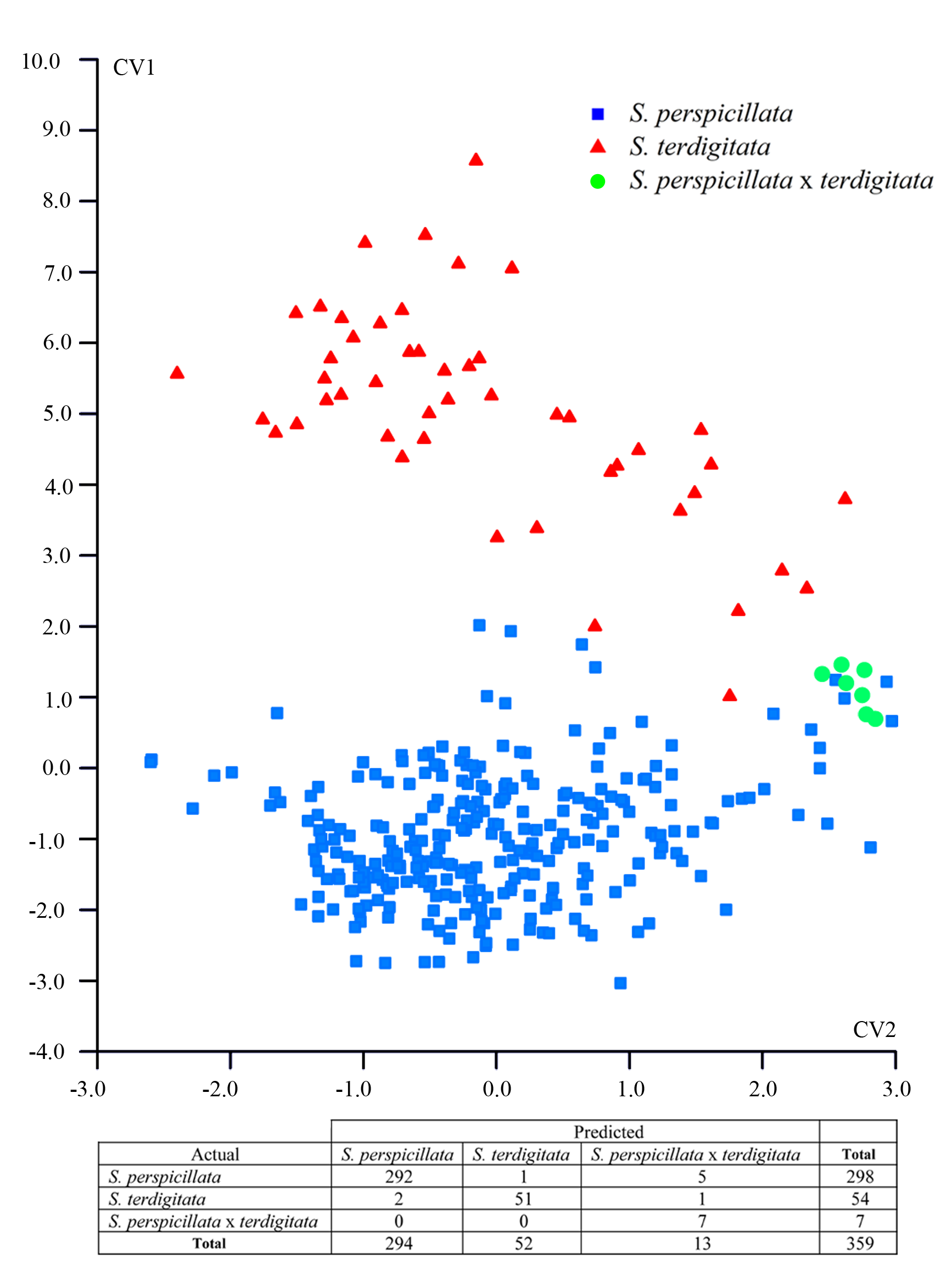


**Supplementary Table S5.** Table reporting the dataset obtained from literature search and personal observations. For the literature reported, the name of the first author and the publication year are reported along with the publication’s name. Coordinates are in WGS84.

**Supplementary File S6.** The set of the nineteen bioclimatic variables considered as candidate predictors (from Worldclim.org), with their codes and explication.

BIO1 = Annual Mean Temperature

BIO2 = Mean Diurnal Range (Mean of monthly (max temp – min temp))

BIO3 = Isothermality (BIO2/BIO7)*100

BIO4 = Temperature Seasonality (standard deviation*100)

BIO5 = Max Temperature of Warmest Month

BIO6 = Min Temperature of Coldest Month

BIO7 = Temperature Annual Range (BIO5-BIO6)

BIO8 = Mean Temperature of Wettest Quarter

BIO9 = Mean Temperature of Driest Quarter

BIO10 = Mean Temperature of Warmest Quarter

BIO11 = Mean Temperature of Coldest Quarter

BIO12 = Annual Precipitation

BIO13 = Precipitation of Wettest Month

BIO14 = Precipitation of Driest Month

BIO15 = Precipitation Seasonality (Coefficient of Variation)

BIO16 = Precipitation of Wettest Quarter

BIO17 = Precipitation of Driest Quarter

BIO18 = Precipitation of Warmest Quarter

BIO19 = Precipitation of Coldest Quarter

**Supplementary File S7.** Model settings. Models built for *S. perspicillata* and *S. terdigitata* were parametrized as follows: General Linear Models (GLM): type = 'quadratic', interaction level=3; Multiple Adaptive Regression Splines (MARS) = type = 'quadratic', interaction level = 3; Generalized Boosting Model (GBM), also known as BRT = number of trees = 10000, interaction depth = 3, cross-validation folds = 10; maxent (MAXENT.Phillips) = maximum iterations = 5000. Ten sets of 1000 pseudo-absences each were generated, selected through a Surface Range Envelope (‘sre’), with a quantile set to = 0.05.
